# Recent Advances and Perspectives on the Health Benefits of Urolithin B, A Bioactive Natural Product Derived From Ellagitannins

**DOI:** 10.3389/fphar.2022.917266

**Published:** 2022-06-22

**Authors:** Peng Chen, Zhiei Guo, Fuchao Chen, Yue Wu, Benhong Zhou

**Affiliations:** ^1^ Department of Pharmacy, Renmin Hospital of Wuhan University, Wuhan, China; ^2^ Department of Pharmacy, Wuhan Fourth Hospital, Puai Hospital, Tongji Medical College, Huazhong University of Science and Technology, Wuhan, China; ^3^ Sinopharm Dongfeng General Hospital, Hubei University of Medicine, Shiyan, China

**Keywords:** anti-aging, antioxidant, biological effect, urolithin B, nutritional agent

## Abstract

Urolithin (Uro) B is a natural compound produced by gut bacteria from ingested ellagitannins (ETs) and ellagic acid (EA), complex polyphenols abundant in foods such as pomegranates, raspberries, blueberries and chestnuts. Uro B has recently garnered considerable attention owing to its wide range of nutraceutical effects and relatively high potency. According to several studies, Uro B prevents the development of hyperlipidemia, cardiovascular disease (CVD) and tumors due to its strong antioxidant and anti-inflammatory properties. Many reviews have systematically summarized the health benefits and pharmacological activities of ETs, EA and urolithins (especially Uro A) while available reviews or detailed summaries on the positive impact of Uro B are rarer. Here, we sought to review the pharmacological activity, mechanism of action, regulation of immune function and its associated diseases and preventive potential of Uro B to elucidate its function as a nutritional agent in humans.

## 1 Introduction

ETs are a complex class of bioactive compounds, mainly comprised of hydrolyzed tannins, found in pomegranates, strawberries, ground elm, blueberries, raspberries, tea, walnuts, chestnuts and mulberries (Cerdá et al., 2005). The consumption of ETs has been consistently associated with positive effects towards many pathologies, including metabolic disorders and diabetes (El-Missiry et al., 2015; Atrahimovich et al., 2018). Even though multiple health benefits have been attributed to ETs, it is known that they have limited bioavailability and consequently unable to reach systemic circulation in considerable concentrations (Cerdá et al., 2003; Cerdá et al., 2004).

ETs have been detected mainly in the gastrointestinal tract compared to other organs because they are metabolized into EA in the stomach, small intestine and colon (Larrosa et al., 2006a; García-Villalba et al., 2020). However, EA is poorly absorbed from the gastrointestinal tract. A study showed that increasing the consumption of ETs did not improve the amounts of EA in circulation (González-Sarrías et al., 2015). EA produced from the hydrolysis of ETs reaches the colon where it is further hydrolyzed and metabolized by gut microbiota into dibenzopyran-6-one derivatives, known as urolithins (Selma et al., 2018). After absorption, these polyphenol-derived metabolites undergo extensive phase-II metabolism to form sulphates and glucuronides, which can be detected in plasma and target systemic tissue up to 3–4 days later, where they might trigger biological effects (González-Sarrías et al., 2017a). In that view, urolithins should be considered as compounds with bioactivity in response to the consumption of ET- or EA-rich foods.

**FIGURE 1:**
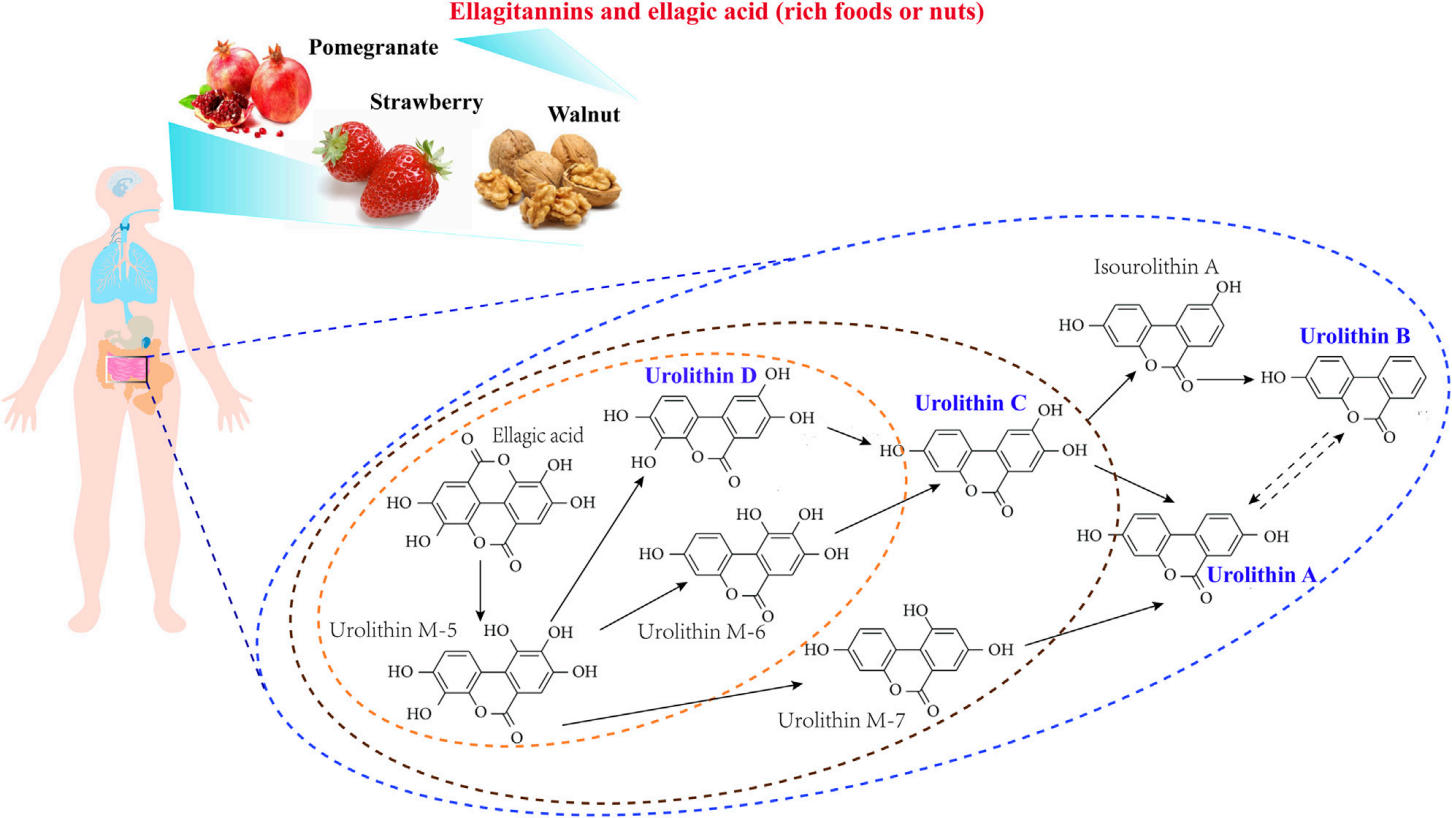
A schematic view of ellagitannins and ellagic acid gut microbiota metabolism pathway.

Nowadays, different Uro intermediates (Figure 1), such as Uro D, C, A, and B have been recognized as metabolites of EA, with Uro A [C_13_H_8_O_4_, 3,8- dihydroxybenzo(c) chromen-6-one] and Uro B [C_13_H_8_O_3_, 3-hydroxybenzo(c)chromen-6-one] serving as the major metabolites present in the gut, while Uro C (3,8,9-trihydroxy-urolithin) and Uro D (3,4,8,9-tetrahydroxy-urolithin) are found at lower concentrations and exert less physiological activity than Uro A or B (Tomás-Barberán et al., 2014; González-Sarrías et al., 2017b; Cortés-Martín et al., 2020; Iglesias-Aguirre et al., 2021). The abundance of UROs (especially Uro A) in the human diet has a direct impact on human health; indeed, several studies have investigated their various biological activities (Andreux et al., 2019; Raimundo et al., 2021).

Uro A is a natural autophagy inducer that effectively enhances mitochondrial function and alleviates endogenous inflammation, ultimately regulating cell health (Ryu et al., 2016). Based on several clinical studies, Uro A can delay aging and improve age-related diseases of the muscle, brain, skin and other organs (Andreux et al., 2019). More interestingly, the biological activity of Uro B has garnered remarkable attention and has been extensively studied in recent years. To date, numerous studies have evaluated various biological activities of Uro B, including its cardiovascular protection, nephroprotection, neuroprotection, anticarcinogenic, antidiabetic, antiobesity, anti-inflammatory, antioxidant and antibacterial properties (Tomás-Barberán et al., 2017; Al-Harbi et al., 2021; García-Villalba et al., 2022).

Based on previous studies, there are several reviews on the health benefits, ramifications and potential applications of Uro A (D'Amico et al., 2021; Toney et al., 2021); however, reviews on Uro B only have not been compiled. Here, the potential role of Uro B as a therapeutic agent, was comprehensively reviewed and the molecular signaling pathways for its biological activities based on cell culture, animals and humans were explored to develop new possible clinical options.

## 2 Mechanisms of Action of Uro B

### 2.1 Anti-Inflammatory Effects

The well-known beneficial effect of Uro B in preclinical models is the attenuation of detrimental inflammatory responses, which can be clinically relevant in the cases of most chronic immune diseases. This chronic phenomenon is called “inflamm-aging,” and contributes to a series of defects in immunological activity, including dysfunction and injury of immune cells and tissues (Franceschi et al., 2018).

The anti-inflammatory effect of Uro B was recently elucidated as a decrease in some inflammatory cytokines, including interleukin-6 (IL-6), Interferonγ(IFN-γ), tumor necrosis factor-α (TNF-α), interleukin-4 (IL-4) and interleukin-1β (IL-1β) in the small intestine of a mouse model of D-galactose (D-gal)-induced aging and HT29 cells (Chen et al., 2021a). The levels of the same cytokines were reduced in RAW264.7 murine macrophages employed in a lipopolysaccharide (LPS) induced inflammatory injury model (Piwowarski et al., 2015). In this model, Nitric oxide (NO) generation decreased by the inhibition of inducible nitric oxide synthase (iNOS) protein and messenger ribose nucleic acid (mRNA) expression. Studies found that treatment of colonic fibroblasts with IL-1β increased prostaglandin E_2_ (PGE_2_) levels and that this response was partially attenuated by 5 or 10 μM Uro B (González-Sarrías et al., 2010a; Giménez-Bastida et al., 2012a). The implicated mechanism of action seemed to be *via* the inhibition of nuclear factor-k-gene binding (NF-kB) activation and mitogen-activated protein kinase (MAPK) in inflammatory colonic cell models (González-Sarrías et al., 2010a). Further studies revealed a reduction in IFN-γ levels in the human THP-1 cell line-derived macrophages stimulated by LPS. Monocyte chemotactic protein-1 (MCP-1) was found to be inhibited by Uro B in kidneys of unilateral ureteral obstruction-induced kidney injury models (Li et al., 2019). Lower levels of fractalkine, a proinflammatory cytokine that influences myocardial function, were found in a streptozotocin-induced rat model of diabetic cardiomyopathy after treatment with Uro B compared with those in the control (Savi et al., 2017). Interestingly, the continued administration of Uro B for 14 days remarkably improved CD68^+^ macrophage infiltration, which was further validated by a decrease in CD68 mRNA expression in rats after myocardial infarction.

Uro B intervention was found to reduce levels of IL-1β, IL-6 and TNF-α in brains of a mouse model of D-gal-induced Alzheimer disease (AD) (Chen et al., 2021b). Owing to its anti-inflammatory and antioxidant properties, Uro B drives neural protection pharmacological activities by inhibiting microglia and astrocyte activation. Prior studies suggest that Uro B reduced glial cell activation in the central nervous system (CNS) and alleviated neuroinflammation (DaSilva et al., 2019; Lee et al., 2019). Notably, the protective effect and neuroinflammatory response of Uro B were goals of neuroimmunology research. In various *in vitro* and *in vivo* models of acute or chronic neurological disorders induced by LPS, Uro B inhibited NO levels and reduced the mRNA expression of pro-inflammatory genes, such as IL-6, TNF-α, IL-1β, iNOS and cyclooxygenase-2 (COX-2) in LPS-stimulated microglia (Lee et al., 2019). Similar findings were obtained with polyinosinic-polycytidylic acid and lipoteichoic acid, which are also stimulators of neuroinflammation in BV2 microglial cells (DaSilva et al., 2019).

Owing to Uro B, the upregulation of the antioxidant, hemeoxygenase-1, *via* nuclear factor erythroid 2-related factor 2 (Nrf2)/antioxidant response element (ARE) signaling in the BV2 cell line strengthened the reactive oxygen species (ROS) scavenging activity by enhancing the nicotinamide adenine dinucleotide phosphate (NADPH) oxidase subunit expression. Other mechanisms of action of Uro B include downregulating the phosphorylation of extracellular signal-regulated kinase (ERK), p38, protein kinase B (AKT) and NF-kB, which are linked to its anti-neuroinflammatory activity. Evidence from LPS-injected mice brains confirmed that Uro B suppressed gliosis and the release of pro-inflammatory factors in the CNS.

Overall, regarding the anti-inflammatory effects of Uro B in systemic tissues, several *in vitro* studies have been performed in different cell models from systemic tissues as well as immune system cells. However, the authors of most of these studies focused mainly on the investigations using free Uro B. Few reported the anti-inflammatory effect of the circulating conjugated metabolites, but lower than their aglycones, against cardiomyocytes for Uro B glucuronide and against human aortic endothelial cells for Uro A and Uro B glucuronides (Giménez-Bastida et al., 2012b; Sala et al., 2015). Sala *et al.* reported that incubation of Uro B glucuronide with neonatal rat ventricular cardiomyocytes cultured in hyperglucidic conditions significantly reduced MCP-1, fractalkine and VEGF expression levels (Giménez-Bastida et al., 2012b). Giménez-Bastida *et al.* demonstrated that approximately 5 μM concentration of UroA-glucuronide and Uro-B-glucuronide inhibited TNF-α-induced migration of human aortic endothelial cells (HAECs) (Sala et al., 2015). These results indicated that the circulating Uro glucuronides may be, at least in part, the molecules responsible for some of the beneficial effects on exerting anti-inflammatory function. Thus, the influence of a mix of circulating metabolites on the inflammatory scenario should be considered and specifically designed intervention studies on the potential protective effects of ET-rich food sources should be investigated.

### 2.2 Antioxidant Properties-Oxidative Stress

Oxidative stress **(**OS) is caused by an imbalance between the excessive production of ROS and a biological system’s ability to readily detoxify ROS (Filomeni et al., 2015). Such imbalances damage multiple cellular processes, such as protein modification (oxidation, carbonylation), physiologically transporting signals *via* a second messenger or deoxyribose nucleic acid (DNA) replication and transcription (Jakubczyk et al., 2020). Increased ROS levels are implicated in many chronic metabolic diseases, e.g., cancer, diabetes, chronic kidney disease and neurodegenerative diseases. Some natural metabolites, such as those with good antioxidant activity, have become a hotspot of current research (Jiang et al., 2021). Based on substantial evidence, Uro B possesses excellent antioxidant properties.

Pfundstein *et al.* measured the antioxidant capacities of Uro B using different *in vitro* antioxidant assays, including the ferric-reducing antioxidant power assay and confirmed its strong antioxidant activity (Pfundstein et al., 2014). However, their findings differed from those of several previous studies, which could be related to the concentration of the compound and the number of times the assay was repeated (Verzelloni et al., 2011). Some studies suggest that Uro B can exert antioxidant activity, protecting cells from oxidative damage. Previously, Lee *et al.* found that Uro B reduced the production of ROS and NADPH oxidase subunit expression and upregulated hemeoxygenase-1 expression *via* Nrf2/ARE signaling to exert powerful antioxidant activity in microglia (Lee et al., 2019). Despite the prominent neuroprotective effects of Uro B, the corresponding evidence is weak, partly due to the lack of physiologically relevant studies using circulating conjugated urolithins that might reach brain tissue. To date, there are no reports on the attenuation of Uro-B glucuronide on the hydrogen peroxide (H_2_O_2_)-induced cytotoxicity in neuroblastoma SH-SY5Y cells, unlike those of Uro A glucuronide (González-Sarrías et al., 2017c).

Furthermore, Qiu *et al.* revealed that the increase in viability of H_2_O_2_-treated T24 cells owing to Uro B was associated with decreased intracellular ROS and malondialdehyde levels and an increase in superoxide dismutase activity (Qiu et al., 2013). In another study, human neuronal cells were treated with 0.5 μM Uro B, which resulted in a significant inhibition in the formation of the end product of advanced glycation, ultimately leading to a good counteraction to mild OS in a conservative *in vitro* experimental model (Verzelloni et al., 2011). As different detection methods were employed, the antioxidant capacity of Uro B also differed. Bialonska *et al.* reported that Uro C (IC_50_ = 0.16 µM) and Uro D (IC_50_ = 0.33 µM) had the highest antioxidant activity, Uro A displayed less significant antioxidant activity (IC_50_ = 13.6 µM) and Uro B, along with all methylated urolithins, did not display any antioxidant activity (Bialonska et al., 2009). The relatively strong antioxidant potency of urolithins reported from earlier studies could be well illuminated as researchers used test methods that are based on physical and enzymatic processes (Djedjibegovic et al., 2020).

## 3 Biological Effects of Uro B on Diseases

This section outlines the biological effects of Uro B on health conditions and diseases, with a particular focus on diseases of the heart, liver, intestine, muscle, kidney and brain. Table 1 summarizes the key points of the pharmacological function of Uro B assayed on different animal models.

**TABLE 1:** Disease state, experimental design and biological activities of Urolithin B *in vivo*.

No.	Condition	Animal model	Dosage	Findings	References
1	Aging	C57BL/6J mice; D-gal-induced aging model	50–150 mg/kg administered once daily, orally for 2 months	Alleviated cognitive deficits and ameliorated brain aging-related conditions	Chen et al. (2021b)
		12-month-old normal aging mice	150 mg/kg once administered once daily, orally for 2 months		
2	Chronic renal damage	Sprague–Dawley rats; Unilateral ureteral obstruction-induced kidney injury	20–80 mg/kg administered once daily, orally for 3 weeks	Ameliorated renal function inflammatory-induced renal fibrosis	Li et al. (2019)
3	Brain diseases	Institute of Cancer Research male mice; Lipopolysaccharide-induced neuroinflammation	50 mg/kg administered once daily *via* intraperitoneal injection for 4 days	Provided therapeutic potential for neuroinflammatory disorders	Lee et al. (2019)
		*C. Elegans*; Aβ-induced Alzheimer disease model	10 μg/ml administered once daily orally from egg stage until death	Protection in post induction of amyloid β_1−42_ induced neurotoxicity and paralysis	Yuan et al. (2016)
4	Cardiovascular disease	C57BL/6 mice; Ligation of the left descending coronary artery-induced myocardial ischemia	2.5 or 5 mg/kg administered once daily *via* intraperitoneal for 2 weeks	Reduced susceptibility to myocardial arrhythmic predisposition after hypoxia	Huang et al. (2022)
		Sprague–Dawley rats; Ischemia reperfusion surgery-induced myocardial ischemia	0.7 mg/kg administered daily *via* intraperitoneal injection for 2 days	Improved cardiac function and reduced susceptibility to ventricular arrhythmias after myocardial ischemia	Gao et al. (2020)
		Sprague–Dawley rats; Ligation of the left descending coronary artery-induced MI	2.5 or 5 mg/kg administered daily *via* intraperitoneal injection for 2 weeks	Protected against superoxide production, apoptotic cell death and myocardial ischemia or reperfusion injury	Zheng et al. (2020)
		Male Wistar rats; Streptozotocin-induced diabetic cardiomyopathy	2.5 mg/kg administered daily *via* intraperitoneal injection for 2 days	Prevented diabetes mellitus associated cardiac dysfunction	Savi et al. (2018)
5	Muscle dysfunction	C57BL/6J mice; Puromycin-induced skeletal muscle atrophy	10 μg administered daily subcutaneously *via* an implanted mini‐osmotic pump for 28 days	Induced muscle hypertrophy in mice and reduced muscle atrophy after the sciatic nerve section	Rodriguez et al. (2017)
	Metabolic diseases	ApoE^−/−^ mice; hyperlipidemia	10 mg/kg daily administered by oral gavage for 14 days	Decreased lipid plaque deposition and cholesterol uptake	Zhao et al. (2019)
		Male Wistar rats; Streptozotocin-induced diabetes mellitus	2.5 mg/kg administered daily *via* intraperitoneal injection for 2 days	Decreased blood glucose levels and body weight	Savi et al. (2018)
		Male Wistar rats; High-fat diet-induced obesity	2.5 mg/kg administered 4 times a week *via* intraperitoneal injection for 4 weeks	Modulated gut microbes related to body weight, dysfunctional lipid metabolism and inflammation	Abdulrahman et al. (2020)
		Male Wistar rats; High-fat diet-induced obesity	2.5 mg/kg administered 4 times a week *via* intraperitoneal injection for 4 weeks	Decrease the bodyweight gain by modulating the composition of gut microbiota	Abdulrahman et al. (2021)
6	Intestinal diseases	C57BL/6J mice; D-gal-induced intestinal injury	50–150 mg/kg administered daily orally for 2 months	Attenuated intestinal immunity function and regulated the composition of gut microbiota	Chen et al. (2021a)
7	Cancer	Nude mice; HepG2 cells injected into the dorsal flank to induce hepatocellular carcinoma model	40 mg/kg administered daily *via* intraperitoneal or subcutaneous injection for 30 days	Decreased average tumor weight and volume	Lv et al. (2019)

### 3.1 CVDs

Although numerous reports in recent studies have revealed the biological activities of Uro A, those on Uro B have mainly been focused on revealing its preventive and protective functions against CVD due to its higher concentration and its sulfate conjugates being abundant in the heart (Huang et al., 2022).

The first cardioprotective effect of Uro B was discovered against a potential trigger for CVD, trimethylamine-N-oxide (TMAO) (Savi et al., 2018). TMAO is a gut microbiota-derived metabolite that is primarily produced from nutrient precursors, such as choline, phosphatidylcholine and L-carnitine under the action of oxidase in the host liver (Hoyles et al., 2021). Cardiac dysfunction and toxicity, including cellular contractility, calcium dynamics and glycogen accumulation, were recovered by urolithin B-glucuronide (Uro B-gluc) in a TMAO-induced rat cardiomyocyte model, demonstrating the good ability of Uro B-gluc in counteracting TMAO-produced cardiomyocyte impairment.

Uro B-gluc might protect against cardiomyocyte damage by increasing the expression of sarco(endo)plasmic reticulum calcium ATPase 2 (SERCA2) and activation of SIRT1, resulting in increased glycolysis through a positive modulation of pyruvate dehydrogenase activity and improved cardiac function. Furthermore, Uro B has been confirmed to improve cardiac function and reduce susceptibility to ventricular arrhythmias after myocardial ischemia, as well as protect against superoxide production and apoptotic cell death during the myocardial ischemia or reperfusion injury (Gao et al., 2020; Zheng et al., 2020).

### 3.2 Metabolic Dysfunction

Several *in vitro* and *in vivo* experiments have been performed to investigate the role of Uro B in metabolic tissues and confirm its protective effects against features of metabolic syndrome, such as atherosclerosis, dyslipidemia, obesity and hyperglycemia. Studies on EA and urolithins in 3T3-L1 adipocytes *in vitro* suggested that the beneficial effects of urolithins against metabolic diseases might be involved *via* the regulation of fat accumulation and inflammation (Cisneros-Zevallos et al., 2020).

Interestingly, in an atherosclerosis-prone apolipoprotein E-deficient (apoE^**−/−**^) mouse model, 10 mg/kg of Uro B displayed protective and healing effects against atherosclerosis (Zhao et al., 2019). This model of transgenic animals exhibited abnormalities in the clearance of triglyceride-rich lipoproteins, which contributed to plasma cholesterol-lipid-calcium deposits in the walls of arteries and the acceleration of atherosclerosis. Treatment with Uro B decreased the lipid plaque deposition in the model group and also inhibited lipid uptake and cholesterol efflux in ox-LDL treated in murine J774 and human THP-1 macrophage cell lines. Further studies revealed that Uro B and Uro B sulfate upregulated the expression of high-density lipoprotein (HDL) receptor scavenger receptor class B type I and adenosine triphosphate (ATP) binding cassette transporter which decreased the level of cholesterol. Moreover, Uro B and Uro B sulfate increased cholesterol efflux from cholesterol laden macrophages to the HDL particle, which is the initial step in reverse cholesterol transport.

In normal-diet fed rats, a daily intraperitoneal injection of Uro B enhanced the metabolic functions of the liver and kidney and promoted the abundance of *Bdellovibrionales* and the growth of *Akkermansia* (Al Khalaf et al., 2021). Owing to the increase in these two important microbes, Uro B has a positive impact on different metabolic diseases and the control of intestinal pathogens. Of note, no treatment-related effects on body weight were observed. In a prior study with a high-fat diet (HFD)-associated obesity model, consecutive intraperitoneal injections of Uro B for 4 weeks reduced the body weight and attenuated visceral adipose tissue mass and OS in rats (Abdulrahman et al., 2020). Moreover, the mRNA expression of LXRα and SREBP1c, which are involved in *de novo* lipogenesis, was downregulated while the expression of PPARα, which caused increased fatty acid oxidation, was upregulated.

Similar lipid-lowering results were reproduced in another model of obesity-associated gut dysbiosis, i.e., rats fed a HFD for a long time, presented with gut dysbiosis; supplementing Uro B regulated the gut microbial community of HFD-fed rats, leading to a decrease in body weight and levels of cholesterol, triglycerides and low density lipoprotein cholesterol (LDL-C) in serum (Abdulrahman et al., 2021). Uro B is a potential therapeutic agent for the management of obesity-associated inflammation and metabolic dysfunction. However, more research is needed to clarify the most appropriate dosage and modeling method.

### 3.3 Skeletal Muscle Function

The proven defensive function of Uro B is not restricted to cardiac muscle. In fact, Uro B has enhanced beneficial effects of androgen in skeletal muscle without any negative impact on the body. Uro B was found to promote the growth and differentiation of cultured C2C12 myotubes, which suggested that Uro B could be a positive regulator of skeletal muscle mass (Rodriguez et al., 2017). Consequently, researchers sought to determine its underlying molecular mechanisms. The incubation effect of Uro B was hypothesized to be based on the regulation of protein synthesis and degradation.

According to *in vitro* data, Uro B stimulated protein synthesis in myotubes *via* an mechanistic target of rapamycin complex 1 (mTORC1)-dependent mechanism, possibly triggered by a decrease in adenosine monophosphate-activated protein kinase (AMPK) activity. In the presence of Uro B, protein degradation was reduced, which may be due to inhibition of the ubiquitin-proteasome system rather than autophagy. Nevertheless, the specific mechanism remained unknown. Data obtained genetically and pharmacologically supported the implication of the androgen receptor in the observed anabolic effect of Uro B *in vitro.* Moreover, during muscle wasting, such as after denervation, Uro B showed interesting properties as it preserved muscle mass. Another study confirmed the protective effect of Uro B against muscular augmentation where 21 days of Uro B gavage decreased fat mass, increase lean body mass and improved muscle function in mice with muscle atrophy by potentially reducing protein degradation through partial inhibition of the ubiquitin-proteasome system activity in muscle, ultimately preventing dexamethasone-induced muscle atrophy in mice (Wang et al., 2018).

Uro B could serve as a positive regulator of skeletal muscle mass; it is conducive to the increase in muscle mass and restricts atrophy (Trombold et al., 2010). Interestingly, Uro B was found to have little effect on the gastrocnemius, which is a powerful muscle that runs from the femur to the Achilles tendon. This contradicts the findings presented in literature regarding the action of dietary quercetin on skeletal muscle growth, which was found to prevent muscle atrophy by regulating the mitochondrial quality control process in the sciatic nerve sections in mice.

### 3.4 Neurodegenerative Diseases of the CNS

Numerous *in vitro* and *in vivo* studies have been performed to define molecular and cellular events underlying beneficial neuroprotective effects of Uro B in various animal models of AD. Yuan *et al.* evaluated the effect of pomegranate peel polyphenols (PPPH) and their intestinal metabolites, urolithins (10 μg/ml), on *Caenorhabditis elegans* using the A*β*_1-42_-induced AD model (Yuan et al., 2016). Of the samples tested, only methylated Uro B caused a significant increase in the mean, maximum and median survival or mobility of *C. elegans* post induction of A*β*_1-42_-induced neurotoxicity and paralysis. However, no significant effect on the maximum or median survival or mobility was noted after treatment with PPPH and Uro A. In a D-gal-induced aging model, Uro B effectively rescued cognitive impairment and improved brain aging-related conditions by activating ERK and phosphoinositide 3-kinase, which promoted neuronal survival, as well as AKT and p44/42 MAPK phosphorylation and activation (Chen et al., 2021b).

As products of gut microbiota metabolism of EA, Uro B contributes to the anti-inflammatory and neuroprotective effects of ETs and their potential anti-apoptotic properties have recently been a topic of interest (DaSilva et al., 2019). DaSilva *et al.* found that relevant concentration of Uro B and its methylated derivatives attenuated neuroinflammation through the inhibition of NO, IL-6, TNF-α, PGE2 and ROS formation in murine BV-2 microglia. González-Sarrías *et al.* revealed that Uro A, Uro B and their methylated derivatives mitigated apoptosis and caspases 3/7 and 9 release from the OS of BV-2 and SH-SY5Y cells *in vitro* ^40^ *.*

Increased monoamine oxidase (MAO) activity affects the inactivation of monoamine neurotransmitters in some neurodegenerative diseases, such as Parkinson’s disease, chronic stress-induced anxiety and depression. Singh *et al.* evaluated the inhibitory effects of Uro A, Uro B, Uro C and EA on MAO activity using recombinant human MAO-A and MAO-B enzymes. Among the tested urolithins, Uro B was proven to be a better MAO-A enzyme inhibitor and with EA (IC value = 0.88 µM), displayed a mixed mode of inhibition (Singh et al., 2020).

### 3.5 Anti-cancer

#### 3.5.1 Breast Cancer

Although Uro A might exert greater antitumor potential than Uro B, the anti-cancer potential of Uro B has been demonstrated in various studies. The pathogenesis of breast cancer is still unclear and conventional treatment options include radiotherapy, chemotherapy, hormone therapy and surgical excision (M Braden et al., 2014; Tray et al., 2019). The proliferation of human breast cancer cells is mainly dependent on estrogen, a hormone that promotes rapid proliferation of breast cancer cells. However, estrogen biosynthesis requires the catalytic action of aromatase (Kumar et al., 2019). Therefore, potential therapeutic strategies for breast cancer cells could be based on the targeted inhibition of this enzyme.

The results of aromatase inhibition in MCF-7aro cells treated with Uro B revealed that the inhibition of both estrogen- and testosterone-induced proliferation in the in-cell assay were significantly improved, indicating that Uro B is a competitive inhibitor of aromatase (Adams et al., 2010). Such findings may be due to the better absorption effect of Uro B at the cellular level. A previous study conducted by Larrosa *et al.* revealed that Uro B is taken up intact in MCF-7 cells and largely metabolized to glucuronide and sulfate conjugates indicating that Uro B has been identified to reach human breast tissue as phase II conjugated (Larrosa et al., 2006b; Ávila-Gálvez et al., 2019). It is worth noting that the data from published studies showed that these conjugated metabolites lacked these activities in the same model even at supraphysiological concentrations in comparison with free unconjugated metabolites (Ávila-Gálvez et al., 2018). Therefore, the potential antiproliferative, cytotoxic, estrogenic or antiestrogenic activities in breast tissue could be mainly governed by the molecular form of each metabolite attained in the tissue.

#### 3.5.2 Prostate Cancer

Prostate cancer (PCa) is one of the most commonly-diagnosed lethal cancers in men in Western countries and the second leading cause of cancer death (Murillo-Garzón and Kypta, 2017). Prostate-specific antigen (PSA) is a serine protease produced by epithelial prostatic cells that has been confirmed to be a biomarker for the diagnosis and screening of PCa (Nguyen-Nielsen and Borre, 2016). However, this approach is markedly concerning as PCa is often over diagnosed and thus overtreated. Several studies have also revealed that PSA is expressed in multiple non-prostatic tissues and its specificity as a biomarker for the pathology of PCa is not very strong (Duffy, 2020). Thus, complete excavation of the characteristics of PSA and identification of new effective preventive strategies for PCa are of particular clinical value and importance.

Beneficial effects of Uro B for PCa therapy have been reported in many studies. In fact, 40 μM Uro B was found to have a strong anti-proliferative activity in androgen dependent LNCaP and independent DU-145 cell lines (Stanisławska et al., 2018). Furthermore, the apoptosis of LNCaP cells was found to be strongly induced by Uro B compared to that of untreated control cells (7.9 ± 0.4% late and 1.3 ± 0.2% early apoptotic cells) (Sánchez-González et al., 2014). Interestingly, only Uro B had an inhibiting effect on PSA secretion. In fact, when the antiandrogens of Uro A or Uro C were mixed, PSA secretion was not affected compared to PSA secretion with the use of 10 μM bicalutamide. These results support the use of Uro B in PCa chemoprevention.

Moreover, to establish which compounds may enter the prostatic tissue to exert a putative chemopreventive effect in this organ, González-Sarrías *et al.* determined whether ETs, EA, urolithins or any other derived conjugate can be found and quantified in the human prostate gland upon consumption of pomegranate juice or walnuts (González-Sarrías et al., 2010b). The results showed that Uro B has been identified in human samples that yield high concentrations in the prostate gland but as phase II conjugates. Furthermore, these findings corroborate the need to design better *in vitro* studies that should focus on the bioactivity of the actual *in vivo* metabolites formed upon consumption of ETs, including Uro B-gluc even at the low concentrations detected.

#### 3.5.3 Colorectal Cancer

Colorectal cancer is a complex disease caused by the interaction of genetic and environmental factors. It is ranked third and fourth in terms of the commonality of cancer and cause of death, respectively. It begins as a polyp in the interior lining of the rectal area of the colon and forms slowly. If left untreated, it metamorphoses into a cancer cell with the ability to be metastasized to other locations in the body. Recently, efforts have been ongoing in searching for anticancer therapeutics of plant origin, focusing on polyphenols and their microbiotic metabolites (Nuñez-Sánchez et al., 2015; Nuñez-Sánchez et al., 2017; Giménez-Bastida et al., 2020).

In colorectal cancer cell lines, Uro B exerts anticancer activity mostly through the promotion of apoptosis and cell cycle arrest (González-Sarrías et al., 2009a; González-Sarrías et al., 2014; Cho et al., 2015; González-Sarrías et al., 2016). In an HT-29 colon cancer cell line, 30 µg/ml Uro B mediated antitumor potentials through the induction of apoptosis by activating caspase 3^75^. The underlying mechanism may be upregulation in the expression of p21 protein and G2/M phase arrest of the cell cycle. In Caco-2 colon cancer cell lines, Uro B caused cell cycle arrest at the S phase (González-Sarrías et al., 2014). Data presented in another study showed that Uro B could simultaneously alter the expression levels of several of these cancer target genes, FGFR2, EGFR, K-Ras, MAP4K4, DUSP6, c-Myc and Fos and regulate fundamental cell processes such as cell growth and cell cycle of colon cancer Caco-2 cells (González-Sarrías et al., 2009a).

Prevention of carcinogenesis may occur by several mechanisms, including the blocking of tumor initiation or the suppression of tumor progression. One way of blocking DNA damage and reducing cell malignization is by modulating phase I and phase II enzymes to enhance the detoxification of carcinogenic compounds (Yu and Kong, 2007). The potential of Uro B in modulating the expression of phase I and phase II detoxifying enzymes has also been studied in both colon cancer cell lines and *in-situ* rat models. According to González-Sarrías *et al*, both Uro-A and Uro B at concentrations achievable *in vivo* (40 µM) induced the expression and activity of CYP1A1 and UGT1A10 (González-Sarrías et al., 2009b). Uro B also significantly induced CYP1B1 and CYP27B1 expressions in Caco-2 cells. These data suggested that Uro B modulated phase I and phase II enzymes in the colon, which may be indicative of tumor initiation blocking mechanisms of action.

#### 3.5.4 Hepatocellular Carcinoma

Hepatocellular carcinoma (HCC) is a common heterogeneous tumor, with a high incidence rate and low 5-year survival rate. Treatment failure may result from low sensitivity to chemotherapy and resistant to chemotherapy or radiotherapy (Sim and Knox, 2018). Developing more effective therapeutic strategies for patients with HCC is of great importance to improve their quality of life (European Association For The Study Of The Liver European Organisation For Research And Treatment Of Cancer, 2012). In recent years, natural products have attracted the attention of many scholars and have high application prospects in the tumor field owing to their satisfactory anti-tumor biological activity.

According to the literature, Uro B has potential antitumor effect, especially on HCC. An *in vitro* study revealed that Uro B could inhibit the growth of HepG2 cells in dose- and time-dependent manners (Kallio et al., 2013). This inhibitory effect may occur through an increase in cell cycle protein expression and apoptosis regulators, such as p53 and p38-MAPK and a decrease in c-Jun phosphorylation. Further *in vitro* results revealed that Uro B inhibited the proliferation of HCC cells induced by cell cycle arrest and apoptosis. Furthermore, Uro B enhanced the expression of phosphorylated β-catenin and blocked its translocation from the nucleus to cytoplasm, ultimately resulting in the inactivation of wingless and int-1(Wnt)/β-catenin signaling (Lv et al., 2019). Uro B was found to suppress tumor growth using a xenograft mouse model. Overall, Uro B inhibited the proliferation of HCC cells *in vitro* and *in vivo* by inactivating Wnt/β-catenin signaling, suggesting that Uro B could be a promising candidate in the development of anticancer drugs targeting HCC.

## 4 Bioavailability

Detectable concentrations of Uro B have been studied in different organs in both animal models and humans. Uro B is bioavailable, reaching the plasma and excreted in the urine (Seeram et al., 2006).

A study provided a single pomegranate juice concentrate containing 387 mg/L of anthocyanins, 1561 mg/L of punicalagins, 121 mg/L of EA and 417 mg/L of other hydrolyzable tannins to 18 human subjects. On the day following juice consumption, Uro A-glucuronide was found in urine samples of 16 subjects and Uro B-gluc in samples of five subjects. The maximum plasma concentrations of total urolithins 6 h after consumption in seven subjects tested for EA metabolites were 0.14 µmol/L and 0.01 µmol/L for Uro A and Uro B, respectively (Seeram et al., 2006). Another study provided a larger dose of pomegranate juice (1 L per day, for 5 days), containing 5.58 g/L of (poly)phenols, including 4.37 g/L of punicalagin isomers. In this report, the circulating urolithins were estimated to reach up to 18.6 μmol/L, with high inter-individual variability (Cerdá et al., 2004).

As for target tissues, a study using rats receiving 23 polyphenol microbial metabolites (total 2.7 µM) intravenously showed that Uro B was sequestered (or metabolized) more rapidly than Uro A in the tissues and it accumulated preferentially in the heart (Gasperotti et al., 2015). Furthermore, another study revealed the presence of aglycones and sulphate forms of Uro B in the pancreas, liver and heart of diabetic rats (Savi et al., 2017). This data is encouraging regarding the bioavailability of Uro B and its use in managing diabetes.

## 5 Pharmacokinetic Characteristics of Uro B

By employing an *in vitro* digestion model (a sequence of oral, gastric and pancreatic digestion, followed by a 24 h fecal fermentation), Mena *et al.* found that the stability of Uro B was markedly higher than that of Uro B-gluc and Uro A (Mena et al., 2017). The researchers compared the initial recovery of the initial quantity at the end of the last (colonic) step among the three substances, which revealed 30 ± 4% for Uro B-gluc (recovered as Uro B, indicating complete deglucuronidation during colonic fermentation) and 16 ± 5% for Uro A. However, this study only proved the bioaccessibility of orally administered Uro isomers; thus, biological data for other routes of administration are yet to be obtained (Piwowarski et al., 2017).

**FIGURE 2:**
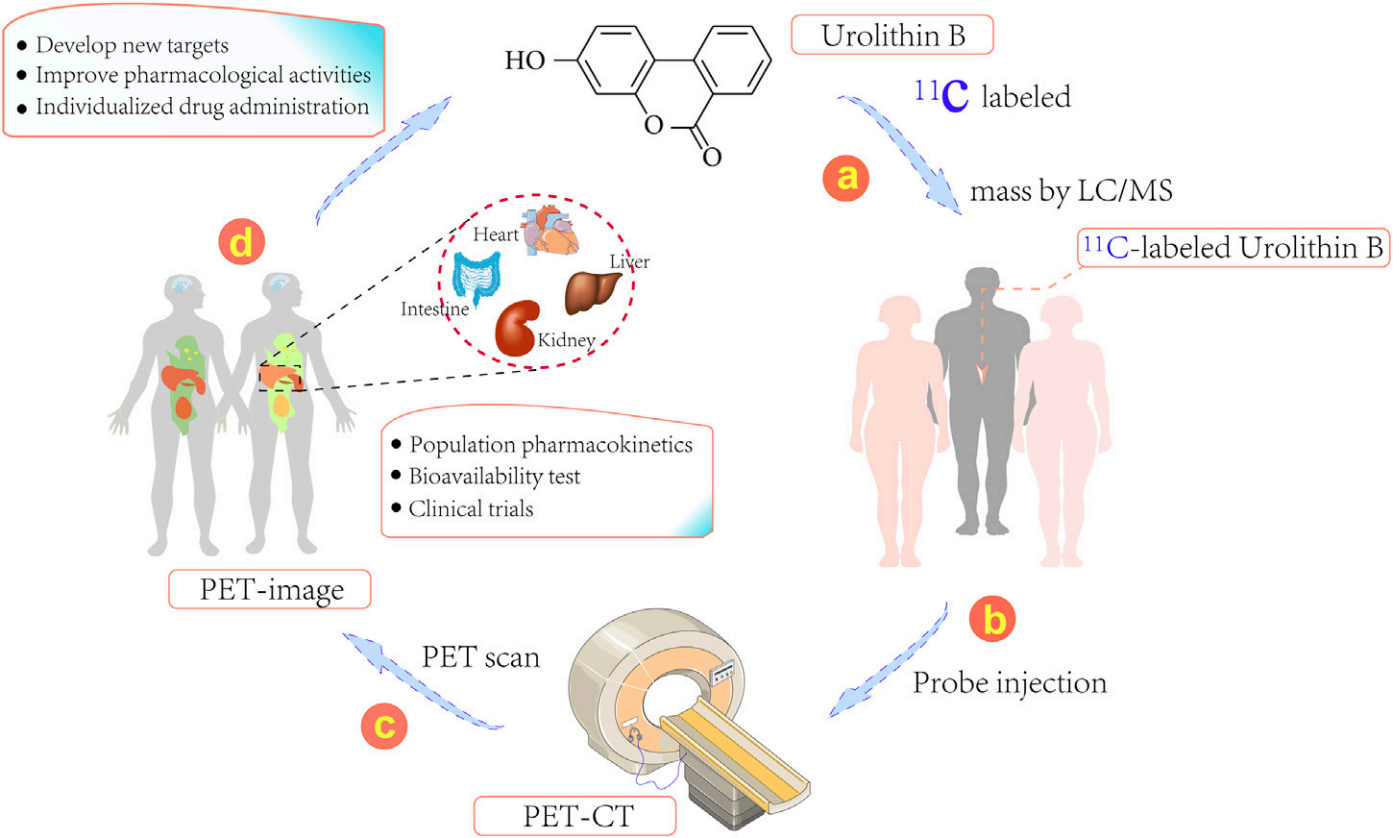
Schematic representation illustrating ^11^C-labeled positron emission tomography (PET) for the development and use of Urolithins. (1) Generation of ^11^C-labeled Urolithins, (2) Injection of ^11^C-labeled Urolithins into patients, (3) PET images effectively captured using the PET-CT imaging system, (4) Discovery of new targets, individualized drug therapy or population pharmacokinetics using this system.

The biological distribution of Uro B is markedly dependent on the protein transporter; thus, cellular uptake of Uro B may involve drug target tissues and its biological distribution in humans can be explored, providing a reliable strategy for studying its mechanism of action in animals (González-Sarrías et al., 2014). The quantitative determination of Uro B in plasma and urine is simple, while intracellular testing is more complex. If tissue is available, the amount of Uro B in a biopsy material can be determined; however, this approach is significantly limited by the risk of biopsy-related complications in target organs of action, such as the liver and kidney (Xian et al., 2021). Of note, to better investigate the distribution of Uro B in organs and tissues, ^11^C-labeled Uro B positron emission tomography (PET) can be applied (Figure 2) (Kim et al., 2019). ^11^C-labeling approach can directly label prototype compound Uro B and avoid the disadvantage of some probes potentially affecting the metabolic distribution and mechanisms of action of compounds by changing their structures.

## 6 Future Prospects

In recent years, nutritional intervention in cancer and CVD has attracted the attention of many scholars. Pomegranates and nuts are a good source of natural polyphenols and are reported as safe and efficacious therapeutic candidates for cancer prevention and individualized treatment (Al-Harbi et al., 2021). Uro B, a gut microbiota metabolite from ET-rich food sources, has strong cardiovascular, anticancer, anti-inflammatory and antidiabetic potential based on existing evidence (Raimundo et al., 2021). However, publications related to this compound are limited to a few *in vitro* reports. Therefore, several *in vivo* pharmacological tests on the anti-tumor activity of Uro B are needed in the future. *In vitro* studies with specific cell models and specific metabolites (type of UROs and conjugates) are useful to unravel mechanisms. In this context, appropriate concentrations and type of metabolites should be carefully chosen to draw relevant conclusions when translating it into what happens *in vivo.*

The bioavailability of Uro B in different cancer types needs to be further studied to predict and evaluate the peak concentration of urolithins and confirm their ability to reach target tissues. Expanding the bioavailability of Uro B will be essential to the development of ideal models that approximate physiological concentrations *in vitro* (Espín et al., 2013). Notably, the solubility of Uro B is a concern, as this property can affect drug delivery. Site-specific drug targeting using particle drug carrier systems must be explored using nanotechnology to enhance the cell absorption and distribution of drugs to improve their pharmacological activity (Jiang et al., 2021).

Finally, the drug sensitivity of phase II metabolites of Uro on cancer cells has not been fully explained at a molecular level (Tang et al., 2021; Vini et al., 2021). In addition, more animal studies with Uro B combined with other anticancer drugs should be conducted. If feasible, the anticancer potential of Uro B must be assessed *via* well-designed human clinical trials. Altogether, current researchers need to address current limitations to enable the development of Uro B and its derivatives into more active molecules.

## 7 Conclusion

Urolithins are metabolites of ET, a natural medicine under the action of intestinal flora in the body. Urolithins are also the material basis for the biological activity of ET *in vivo*. In this review, the antioxidant, anti-inflammatory, anti-tumor and other biological activities of Uro B were discussed and its poor solubility and poor physical and chemical properties that limit its biological activity were presented. Owing to the diversity and complexity of the mechanisms of action of Uro B, caution must be exercised when *in vitro* studies or animal models are employed to predict cellular mechanisms. The intensity of the action of Uro B depends on its uptake by target cells. However, the biological distribution of drugs, such as ^11^C-PET, in the human body can help identify target tissues and optimize the dose in mechanism studies. In the era of precision medicine, continued exploration of the involvement of Uro B in biochemical interactions is important. Such assessments may facilitate the discovery of new therapeutic options, other indications for prescribing Uro B and complete understanding of its molecular processes to enable its use to treat cancer, diabetes and age-related diseases.
